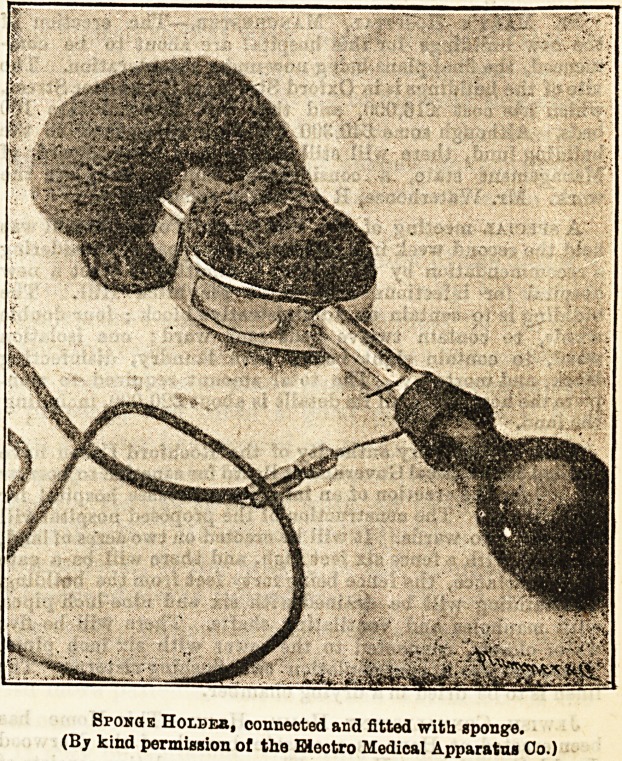# New Drugs. Appliances, and Things Medical

**Published:** 1893-05-06

**Authors:** 


### NEW DRUGS, APPLIANCES, AND THINGS MEDICAL.

[All preparations, appliances, novelties, etc., of which a notice is desired, should be sent for the Editor, to care of The Manager, 428, Strand, London, W.C.]

### NEW SPONGE-HOLDING ELECTRODE.

The Electro-Medical Apparatus Company, Trafalgar Chambers, 36, St. Martin's Lane, W.C., have lately introduced

Sponge Holder, connected and fitted with sponge.
(By kind permission of the Electro Medical Apparatus Co.)

a new sponge-holding electrode, which can claim several advantages over those previously in use. Nurses who are called upon to apply electricity to their patients sometimes find it difficult to secure electrodes which shall be at the same time sufficiently soft and sufficiently firm. Electrodes padded with flannel or wash leather are often too harsh for use upon delicate skins, and sponges, while satisfactory enough in this respect themselves, are seldom so secure in their holders as to allow of the stroking movements necessary. The L.K. sponge-holder shown in our illustration meets these difficulties in a thoroughly satisfactory manner, for it not only will hold almost any sized sponge lightly and firmly, but it is so constructed that the sponge can in a second or two be removed for cleaning purposes or renewal, and as quickly replaced. The electrical contact is also good, so that no shocks or electrical irregularities can arise from its use. It will be seen that this is a great improvement upon those holders where the sponge is firmly fixed in and can only be removed at the expense of both trouble and time. Nurses who are constantly using appliances of this description will be very grateful, we feel sure, for so really useful an invention. The accompanying drawing shows the sponge-holder ready for use, connected and fitted with sponge. The self-evident simplicity of this sponge-holder is a strong point in its favour, and its reasonable price will be an additional recommendation. It is nickel plated, with ebonite handle, and its cost is 5s.

## Figures and Tables

**Figure f1:**